# A Synergy of Ultrasound and Bronchoscopy in Enhancing Safety in Percutaneous Tracheostomy Procedures: A Systematic Review and Meta-Analysis

**DOI:** 10.7759/cureus.80708

**Published:** 2025-03-17

**Authors:** Spandan Rajuri, Kiran Kumar, Kalyan Rakam

**Affiliations:** 1 Critical Care Medicine, Cleveland Clinic Abu Dhabi, Abu Dhabi, ARE; 2 Critical Care Medicine, Al Dhafra Hospital, Abu Dhabi, ARE; 3 Critical Care Medicine, Asian Institute of Gastroenterology (AIG) Hospitals, Hyderabad, IND

**Keywords:** bronchoscopy, intensive care unit, percutaneous tracheostomy procedures, safety, ultrasound

## Abstract

Bronchoscopy-guided percutaneous dilatational tracheostomy (BPDT) and ultrasound-guided percutaneous dilatational tracheostomy (USPDT) are widely employed techniques. However, USPDT provides better vascular mapping and reduces bleeding risk, while BPDT offers better tracheal entry and fewer airway complications. Their comparative efficacy and safety were systematically evaluated, with special consideration for high-risk patients, including obese and critically ill individuals with complex airway anatomy. Following the Preferred Reporting Items for Systematic Reviews and Meta-Analyses (PRISMA) guidelines, an in-depth literature search was conducted in Embase, PubMed, Scopus, and Cochrane Library, focusing on adult patients undergoing percutaneous tracheostomy with USPDT, BPDT, or both. Quality assessment indicated that most studies exhibited a low risk of bias, though concerns regarding randomization and selective reporting were noted in some cases. A meta-analysis was conducted using pooled effect sizes, procedural success rates, complication rates, and heterogeneity (I²), applying a random-effects model. Ten studies involving 1,069 patients were analyzed. The pooled analysis demonstrated a moderate positive association between USPDT and BPDT in improving procedural success and reducing complications (CI: 0.41 to 0.55, standardized mean difference = 0.48, 95%, p < 0.05). However, significant heterogeneity (I² = 72.95%) was observed, likely due to variations in study design and patient populations. USPDT and BPDT are both practical and safe for percutaneous tracheostomy, with unique advantages for different clinical scenarios. The findings support a hybrid approach integrating both modalities to enhance procedural safety and efficiency, particularly in high-risk populations. Future large-scale trials should focus on reducing heterogeneity, assessing long-term outcomes, and improving cost-effectiveness to establish best-practice guidelines for broader clinical implementation.

## Introduction and background

Percutaneous tracheostomy (PT) has taken its place as an integral procedure in care units of high incentive for the patients demanding prolonged mechanical respiration with the advantages of shorter procedural times, lower infection rates, and cost-effectiveness over traditional surgical tracheostomy [1]. Though not without risks, such as bleeding, misplacement of the tracheostomy tube, and inadvertent damage to nearby structures, these types of procedures are more challenging in patients with complex anatomy or comorbidities [2]. Advanced tools like ultrasound and bronchoscopy have been introduced into PT practice to control these risks and increase procedural precision. Detailed real-time visualization of the anterior neck anatomy is provided by ultrasound, while bronchoscopy guarantees accurate guidance of the tracheal intubation and confirmation of the tracheostomy tube position [3,4].

The combined use of ultrasound and bronchoscopy represents a synergistic approach that offers advantages from combining the strengths of both modalities: cross-verification of anatomic landmarks and procedural steps in real time [5]. There is minimal room for error in complex clinical situations, such as distorted anatomy, previous neck surgery, or emergency, and this dual modality approach is particularly valuable [6]. There is preliminary evidence that ultrasound and bronchoscopy can produce markedly enhanced procedural safety, lower complications, and better overall outcomes. Despite this, existing studies using these tools tend to be isolated, and the limits of using this set of tools together have not been extensively explored. Additionally, variability in the procedural methods, patient populations, and operator experiences further hinder the ability to make definitive conclusions [7,8].

This systematic review and meta-analysis aims to fill this gap and make systematic synthesis available on clinical proof of synergistic effects of ultrasound and bronchoscopy in PT. The resulting evaluation will assess the impact of the group as a whole on procedural safety, efficacy, and patient outcomes so that best practices can be used to establish evidence-based recommendations for clinical implementation. At the same time, this work attempts to provide a robust basis for adopting this dual modality approach and, to that end, to increase the safety, precision, and progress of PT procedures in critically ill patients by initially consolidating insights from disparate studies.

## Review

Methodology

This systematic review was conducted using the Preferred Reporting Items for Systematic Reviews and Meta-Analyses (PRISMA) guidelines, using a rigorous methodology based on the PICOS (population, intervention, comparison, outcome, study design) criteria, which defined the study’s scope and inclusion parameters. The target population comprised adult patients aged 18 years and older who underwent percutaneous tracheostomy (PT) procedures, ensuring a focused investigation of this demographic. The intervention under study was the combined use of ultrasound and bronchoscopy, emphasizing their synergistic role in enhancing procedural safety, precision, and overall efficacy.

Data Sources and Search Strategy

Literature research was made to identify relevant publications that have evaluated the effect of ultrasound and bronchoscopy on PT. In 2000, all precoded and bibliographic keywords and the MeSH were utilized to perform the research in the four major electronic databases: Embase, PubMed, Cochrane Library, and Scopus. A search strategy of PRISMA guidelines was followed and included “percutaneous tracheostomy”, “ultrasound-guided tracheostomy”, “bronchoscopy-guided tracheostomy”, “safety”, “complications”, and “efficacy”. It was searched through with the application of Boolean operators (AND, OR) to make search results more refined. Filters were implemented to include cohort studies, randomized controlled trials, and case-control studies (Table 1).

**Table 1:** Search strategy across databases.

Database	Search terms used	Filters applied	Truncations/syntax
PubMed	("percutaneous tracheostomy" OR "percutaneous dilational tracheostomy" OR "PDT") AND ("ultrasound-guided tracheostomy" OR "bronchoscopy-guided tracheostomy") AND ("complications" OR "safety" OR "efficacy")	Human studies, adults (≥18 years), English language, 2000–2024	Truncation () for “tracheostom”; Boolean operators (AND/OR)
Google Scholar	allintitle: ("percutaneous tracheostomy" OR "ultrasound tracheostomy" OR "bronchoscopy tracheostomy") AND ("complications" OR "outcomes" OR "success")	First 200 relevant results screened, English language, 2000–2024	Exact phrase search (“”); Boolean operators (AND/OR)
Embase	('percutaneous tracheostomy'/exp OR 'dilational tracheostomy'/exp) AND ('ultrasound-guided' OR 'bronchoscopy-guided') AND ('outcomes' OR 'safety' OR 'success')	Human studies, adults (≥18 years), English language, 2000–2024	Proximity search (NEAR/3) for “tracheostomy NEAR/3 ultrasound”; Boolean operators (AND/OR)
Cochrane Library	("percutaneous tracheostomy" OR "dilational tracheostomy") AND ("bronchoscopy" OR "ultrasound" OR "dual-modality")	Cochrane Reviews, Trials; Language: English; 2000–2024	Exact phrase search (“”); Boolean operators (AND/OR)

Additional manual screening of reference lists from key studies was performed to ensure the inclusion of all relevant literature. The search was restricted to English-language publications. Duplicate records were removed before further screening.

Inclusion and Exclusion Criteria

The inclusion and exclusion criteria were established using the PICOS structure to ensure a systematic selection of studies relevant to the research objective (Table 2).

**Table 2:** Inclusion and exclusion criteria based on the PICOS framework. PICOS: population, intervention, comparison, outcome, and study design; PT: percutaneous tracheostomy; USPDT: ultrasound-guided percutaneous dilatational tracheostomy; BPDT: bronchoscopy-guided percutaneous dilatational tracheostomy; RCTs: randomized controlled trials.

Criteria	Inclusion	Exclusion
Population	Adult patients (≥18 years) undergoing PT with a dual-modality approach (USPDT + BPDT).	Pediatric patients (<18 years).
Intervention	Studies assessing procedural safety, complication rates, or success rates of PT using both ultrasound and bronchoscopy.	Studies using only ultrasound or only bronchoscopy without a combined approach.
Comparison	Studies comparing the dual-modality method to single-modality PT or surgical tracheostomy.	Studies without a relevant comparator or focusing on unrelated airway procedures.
Outcomes	Studies reporting safety outcomes (e.g., complication rates), procedural success, and patient outcomes.	Studies without outcome data or lacking detailed safety/effectiveness measures.
Study design	RCTs, cohort studies, and case series.	Editorials, reviews, book chapters, expert opinions, in vitro, and animal studies.
Language	Articles published in English.	Non-English publications.

Data Extraction and Synthesis

Two reviewers (AA and BB) independently extracted data using a standardized and pre-defined data extraction form. Among the extracted data, varied were study characteristics like author(s), publication year, design study, sample size, procedural details, patient demographics, and important outcomes like safety, rates of complication, etc. The follow-up duration was also noted. In discrepancies between AA and BB, resolution was achieved through discussion or turning to a third reviewer (CC). The corresponding authors were contacted for further clarification when data were incomplete or unclear.

Quality Assessment

Risk of bias: For randomized controlled trials, the Cochrane Risk of Bias 2 tool was used, which included key domains of the randomization process, deviations from the intended interventions, and measurement of outcomes [9]. Observational studies were based on the Newcastle-Ottawa Scale, which evaluated the selection, comparability, and outcome assessment aspects. AA and BB independently conducted risk of bias assessments, and any discrepancies were resolved with consensus. The risk of bias in the findings and the synthesis of evidence was taken into account in the interpretation of the findings and preparation of the conclusions, taking into account the most reliable and robust available data very carefully [10].

Publication bias: Egger’s test and funnel plots were used to assess publication bias. Funnel plots were inspected for asymmetry, which could indicate selective reporting of positive results. Egger’s regression test was performed to evaluate quantitatively small-study effects, with a significant p-value suggesting possible bias. When publication bias was detected, the trim-and-fill method was applied to estimate and adjust for the missing studies, ensuring a more accurate effect size calculation [11].

Statistical Analysis

Statistical data were gathered for meta-analysis, including mean differences, risk ratios, confidence intervals, and p-values. A random-effects model was used for quantitative synthesis due to anticipated variability among studies. Heterogeneity was assessed using the I² statistic with predefined thresholds to determine the degree of inconsistency. Subgroup analyses were conducted based on patient risk factors such as obesity and critical illness.

Results

Article Selection

**Figure 1:**
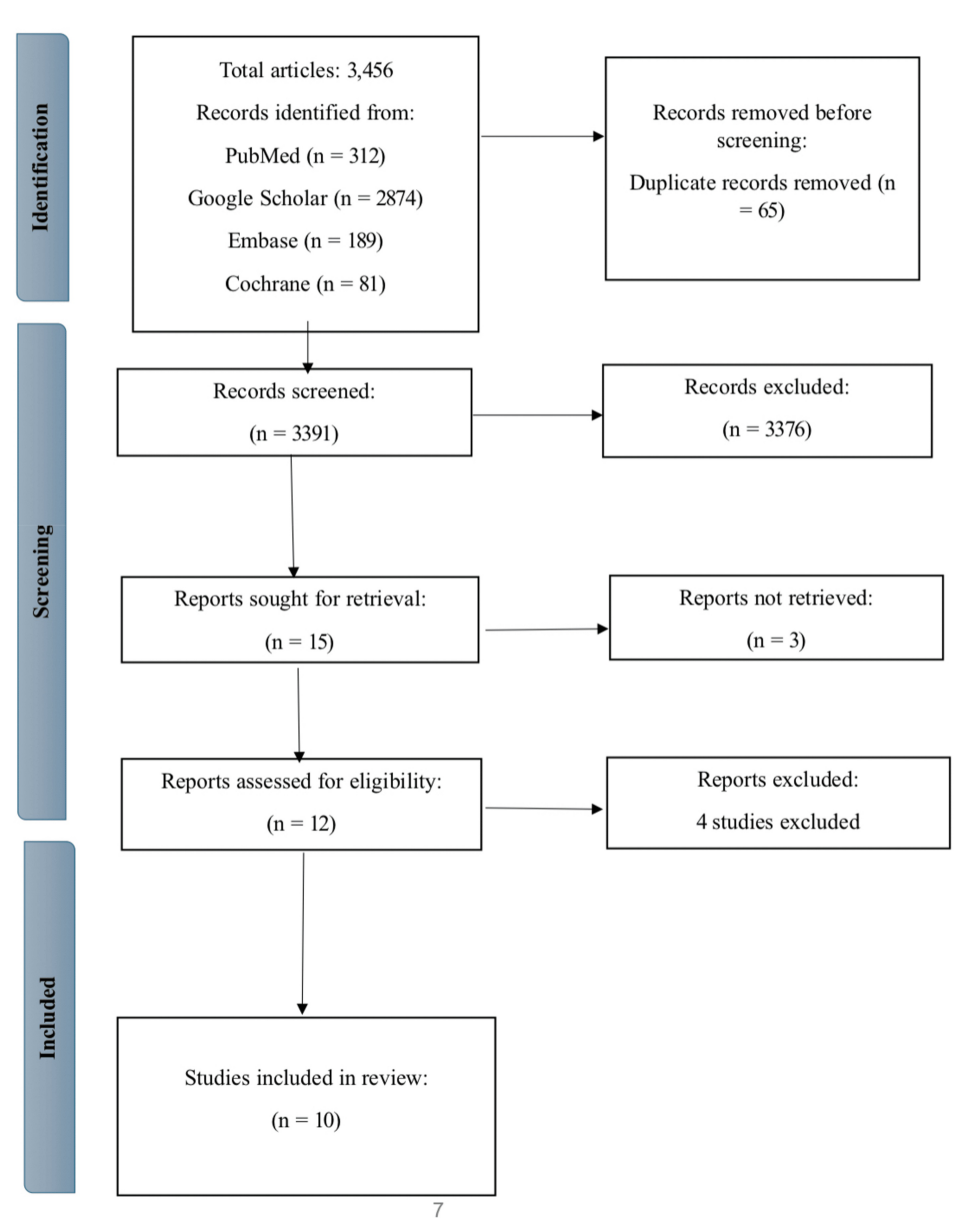
Identification of studies from databases and register.

The systematic search yielded 3,456 articles across various databases and sources, including PubMed (n = 312), Google Scholar (n = 2,874), Embase (n = 189), and Cochrane (n = 81). After removing 65 duplicates, 3,391 articles remained for the initial screening phase. During the title and abstract screening process, 3,376 articles were omitted for failing to meet the inclusion criteria. Three of the remaining 15 articles could not be retrieved due to access restrictions. Consequently, 14 articles underwent a full-text review for eligibility. Four studies were excluded [12-15] for reasons outlined in Table 3, leaving 10 studies eligible for inclusion in the final analysis. A detailed visualization of the study selection process is given in the PRISMA flow diagram in Figure 1.

**Table 3:** Reasons for exclusion of the studies.

Study	Reason for Exclusion
Gunawan et al. (2024) [12]	Study involving pediatric subjects only
Yaghoubi et al. (2020) [13]	Retrospective design with incomplete data
Chen et al. (2022) [14]	Use of AI without bronchoscopy; article retracted
Berlanga-Macías et al. (2022) [15]	The traditional method is used rather than ultrasound guidance in procedure

Table 4 compares ultrasound-guided percutaneous dilatational tracheostomy (USPDT) and bronchoscopy-guided percutaneous dilatational tracheostomy (BPDT), highlighting their strengths and limitations [16-25]. While each technique has distinct advantages, both also present notable challenges. However, combining these methods could enhance procedural accuracy, reduce complication rates, and improve overall patient outcomes. A hybrid approach leveraging the complementary advantages of ultrasound and bronchoscopy provides a more comprehensive strategy, particularly for complex or high-risk cases.

**Table 4:** Characteristics of the included studies. RCT: randomized controlled trial; PDT: percutaneous dilatational tracheostomy; USPCT: ultrasound-guided percutaneous tracheostomy; BPCT: bronchoscopy-guided percutaneous tracheostomy; USPDT: ultrasound-guided percutaneous dilatational tracheostomy; BPDT: bronchoscopy-guided percutaneous dilatational tracheostomy.

Author & year	Country	Study design	Sample size (N)	Patient population	Age (mean ± SD)	Intervention	Procedural success rate & procedure duration	Outcomes measured (including statistical data)	Limitations
Kollig et al. (2000) [16]	Estonia	Observational study	72	ICU patients requiring tracheostomy	52 years (mean)	Percutaneous dilatational tracheostomy (PDT) with ultrasound and bronchoscopic guidance	98.6% success rate; the procedure duration was not explicitly mentioned	Reduced complications, increased safety with ultrasound and bronchoscopy, cost-effective	Small sample size, single-center study, lacks long-term follow-up on complications [24]
Chacko et al. (2012) [17]	India	Observational study	177	ICU patients requiring PDT	Not specified	Ultrasound-guided PDT with and without bronchoscopy	100% (except 1 case requiring open tracheostomy) USPDT 10.7 min vs. BPDT 13.9 min (p < 0.0001)	Oxygen desaturation <90%: USPDT 3.7% vs. BPDT 16.8% (p = 0.006). Minor bleeding: 6.2%, no significant difference between the groups	Retrospective, single-center study. Operator discretion influenced bronchoscope use. Lack of long-term follow-up for all patients [20]
Majid et al. (2014) [18]	USA	Retrospective case series	35	High-risk ICU patients (obesity, prior neck surgery, airway anomalies, coagulopathy)	66 ± 11	Rigid bronchoscopy-guided PDT (RBG-PDT)	Success: 100%; Duration: 32 ± 10 min	No major periprocedural complications, minor bleeding in 2 cases (controlled with suction/epinephrine), 2 cases of transient airway loss (quickly resolved)	Single-center study, lack of direct comparison with flexible bronchoscopy-guided PDT, no cost-effectiveness analysis [25]
Ravi & Vijay (2015) [19]	India	RCT	74	Critically ill ICU patients, including obese patients	USPCT: 62 ± 1.6, BPCT: 58 ± 1.2	Ultrasound-guided percutaneous tracheostomy (USPCT)	Success: 100%; Duration: 12 min (9–14)	Complication rate lower in USPCT (32.2%) vs. BPCT (75%) (p < 0.05), USPCT had fewer minor bleeding cases (p < 0.05), no surgical conversions or deaths	Single-center study, small sample size, limited generalizability [16]
Gobatto et al. (2016) [20]	Brazil	RCT (noninferiority)	118	Mechanically ventilated ICU patient	USPCT: 49.9 ± 16.6, BPCT: 46.9 ± 18.6	Ultrasound-guided percutaneous dilational tracheostomy (USPDT)	Success: 98.3%; Duration: 11 min (7–19)	No major complications in either group, with minor complications higher in USPDT (33.3%) vs. BPDT (20.7%) (p = 0.122), USPDT noninferior to BPDT (90% CI: −5.57 to 5.85)	Single-center study, unblinded outcome assessment, no long-term follow-up [17]
Pilarczyk et al. (2016) [21]	Germany	Retrospective study	93	Thoracic transplant recipients requiring prolonged mechanical ventilation	49.5 ± 11.2	Bronchoscopy-guided PDT using the Ciaglia Blue Rhino technique	Success: 100%; Duration: Not specified	No major complications, moderate bleeding in 3 patients, 48.4% weaned from ventilation, 51.6% died (sepsis, multi-organ failure, transplant failure)	Retrospective study, single-center, lack of control group, no direct comparison with ultrasound guidance [23]
Shen et al. (2019) [22]	China	RCT	90	Mechanically ventilated ICU patient	62 ± 15	Fiber optic bronchoscopy-guided PDT (FOB-PDT)	Success: FOB-PDT (93.3%) vs. PDT (64.4%); Duration: FOB-PDT (9.8 ± 1.2 min) vs. PDT (12.9 ± 1.1 min) (p < 0.05)	FOB-PDT had a lower total complication rate (20%) vs. PDT (40%) (p < 0.05), lower major bleeding, and higher first-pass success	Single-center study, lack of ultrasound for vascular assessment, limited generalizability [19]
Tariparast et al. (2022) [23]	Germany	RCT	46	ICU patients requiring PDT	62 ± 13	Single-use bronchoscope for BPDT	Success: Noninferior; Duration: 10 ± 6 min	No significant differences in ventilation quality, noninferior visualization, higher cost for single-use bronchoscopes	Single-center study, small sample size [18]
Nazir et al. (2022) [24]	Pakistan	RCT	52	Obese ICU patients (BMI ≥30 kg/m²) requiring PDT	Group A: 48 ± 13, Group B: 47 ± 14	Ultrasound-guided PDT (Group A) vs. bronchoscopy-guided PDT (Group B)	Success: Group A (56% single puncture), Group B (41% single puncture); Duration: Group A (8–10 min) vs. Group B (12–15 min)	Group A had fewer intra-procedural complications (6.6%) vs. Group B (21.4%), lower bleeding rates, and fewer multiple punctures	Single-center study, small sample size, lack of long-term follow-up [21]
Carboni Bisso et al. (2023) [25]	Argentina	Prospective observational study	312	ICU patients undergoing bronchoscopy-guided PDT, including COVID-19 and non-COVID-19 patients	66 (IQR 54–74)	Bronchoscopy-guided PDT	Success: 100%; Duration: Anesthesia time: 12 min (10–15), Surgical time: 5 min (4–9)	Oxygen desaturation: 20.8% (COVID-19: 27.3% vs. non-COVID-19: 11.6%, p < 0.01). Minor complications: 7.37%. No major complications. No need for conversion to open tracheostomy	Single-center study, lack of comparison with ultrasound-guided technique, limited generalizability [22]

In India, Ravi & Vijay [19] conducted a prospective, randomized, controlled trial that demonstrated the distinct benefits of each approach. The study found that ultrasound-guided PT resulted in significantly fewer complications and a shorter procedure time than bronchoscopy-guided PT in critically ill obese patients. However, USPDT lacks continuous airway visualization, which prevents complications during needle insertion. BPDT, while providing real-time internal guidance, is associated with higher procedural costs and a greater risk of bleeding and ventilation disturbances. A combined approach could optimize safety and procedural efficiency by using ultrasound for anatomical precision in preoperative mapping and bronchoscopy for continuous airway visualization during the procedure.

Gobatto et al. [20] conducted a randomized noninferiority trial in Brazil, demonstrating that USPDT was as effective as BPDT in preventing significant complications and ensuring overall procedural efficacy. However, the study found that minor complications were slightly more frequent with USPDT, likely due to its limited capability to provide direct airway visualization. BPDT effectively guided the internal aspects of the procedure but was limited in detecting external vascular structures and incurred higher procedural costs due to specialized equipment and personnel. Using ultrasound for vascular mapping and optimal puncture site selection, followed by bronchoscopy for real-time procedural guidance, could minimize the risks associated with each technique and improve overall safety.

Tariparast et al. [23] found that single-use bronchoscopes were non-inferior to reusable ones in terms of visualization and ventilation quality, though they incurred higher costs. Shen et al. [22] in China provided further evidence supporting the complementary nature of these techniques. Their study, which compared fiber optic bronchoscopy-guided tracheostomy (FOB-PDT) with standard percutaneous dilatational tracheostomy (PDT), found that FOB-PDT had a higher success rate, lower complication rate, and shorter procedural duration. However, the absence of ultrasound in this study meant that complications related to unrecognized vascular structures were not addressed. This reinforces the potential benefits of combining ultrasound for anatomical assessment before the procedure with bronchoscopy for real-time intraoperative monitoring to optimize patient safety and procedural success. Chacko et al. [17] reported that USPDT significantly reduced oxygen desaturation rates (<90%) compared to BPDT (3.7% vs. 16.8%, p = 0.006), with not much difference in minor bleeding rate.

Nazir et al. [24] further emphasized the benefits of a combined approach. Their findings showed that ultrasound-guided procedures had lower complication rates and shorter procedure times than bronchoscopy-guided approaches. However, USPDT alone was insufficient for continuous airway monitoring, which is crucial in preventing complications during tube placement. BPDT, while offering real-time monitoring, faced challenges related to intraoperative hemorrhage and visualization limitations. A hybrid approach using ultrasound for initial site selection and bronchoscopy for precise needle and tube placement could bridge these gaps and enhance overall procedural safety.

Carboni Bisso et al. [25] conducted a prospective observational study in Argentina, focusing on BPDT in COVID-19 and non-COVID-19 patients. Although BPDT was successful in all cases, it was associated with an increased risk of oxygen desaturation in COVID-19 patients due to aerosol generation, heightening the risk of viral transmission. Ultrasound can complement bronchoscopy in infectious disease settings by minimizing airway manipulation, enhancing procedural safety, reducing exposure risks, and improving overall efficiency.

Pilarczyk et al. [21] demonstrated the effectiveness of bronchoscopy-guided PDT in thoracic transplant patients, reporting no significant complications and successful ventilation weaning in 48.4% of cases. However, 51.6% died due to underlying conditions. Kollig et al. [16] emphasized the cost-effectiveness and safety of ultrasound and bronchoscopy-guided PDT, with a 98.6% success rate and no significant complications. Majid et al. [18] highlighted the feasibility of rigid bronchoscopy-guided PDT in high-risk patients, achieving a 100% success rate with minimal complications.

These studies highlight the strengths and limitations of both USPDT and BPDT. USPDT excels in identifying anatomical structures, reducing vascular injuries, and shortening procedure duration, yet it lacks the continuous airway visualization crucial for real-time monitoring. BPDT, while providing superior internal guidance, is associated with higher costs, ventilation-related risks, and a reliance on specialized equipment and personnel. A combined strategy leveraging ultrasound for anatomical mapping and bronchoscopy for real-time visualization could enhance procedural safety, reduce complications, and improve patient outcomes in diverse clinical settings.

Quality assessment

Risk of Bias

**Figure 2:**
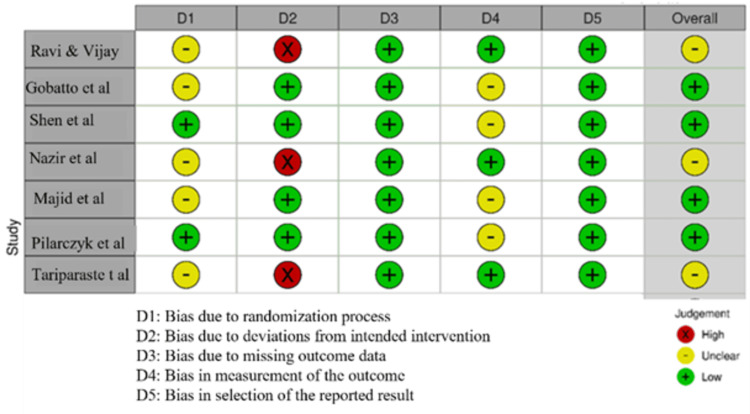
Intra-review bias assessment using the Risk of Bias (RoB) in the trials (for RCTs). Ravi & Vijay (2015) [19], Gobatto et al. (2016) [20], Shen et al. (2019) [22], Nazir et al. (2022) [24], Majid et al. (2014) [18], Pilarczyk et al. (2016) [21], Tariparast et al. (2022) [23]. RCTs: randomized controlled trials.

**Figure 3:**
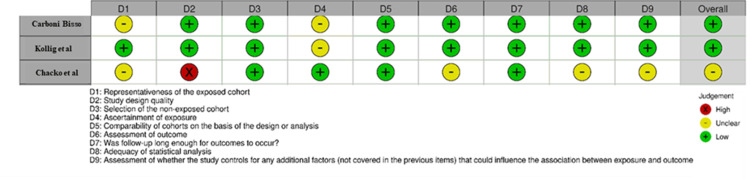
Intra-review bias assessment using the Newcastle-Ottawa Scale (NOS) in the trials (for observational studies). Carboni Bisso et al. (2023) [25], Kollig et al. (2000) [16], Chacko et al. (2012) [17].

The Risk of Bias (RoB) tool and the Newcastle-Ottawa Scale (NOS) were used to assess the risk of bias and the study quality for observational studies (Figures 2, 3). Most studies had a low risk of bias overall, but some had concerns related to their methods. Most studies exhibited a low risk of bias across key domains, particularly in outcome measurement (D4) and handling of missing data (D3), ensuring reliable findings. However, Nazir et al., Ravi & Vijay, and Tariparast et al. had unclear risk (-) in randomization (D1), raising concerns about potential selection bias. Additionally, Ravi & Vijay, Nazir et al., and Tariparast et al. showed high risk (X) in deviations from intended intervention (D2), suggesting significant deviations that might have influenced results. While most studies had low risk (D5) in reported result selection, Gobatto et al., Majid et al., and Tariparast et al. had unclear risk (-), indicating possible selective reporting bias [26]. All studies had strong comparability and exposure ascertainment for NOS assessment, ensuring reliable outcome evaluation. Chacko et al. was the weakest due to its high-risk study design, while the other two studies had minor concerns about selection bias [27].

Publication Bias

**Figure 4:**
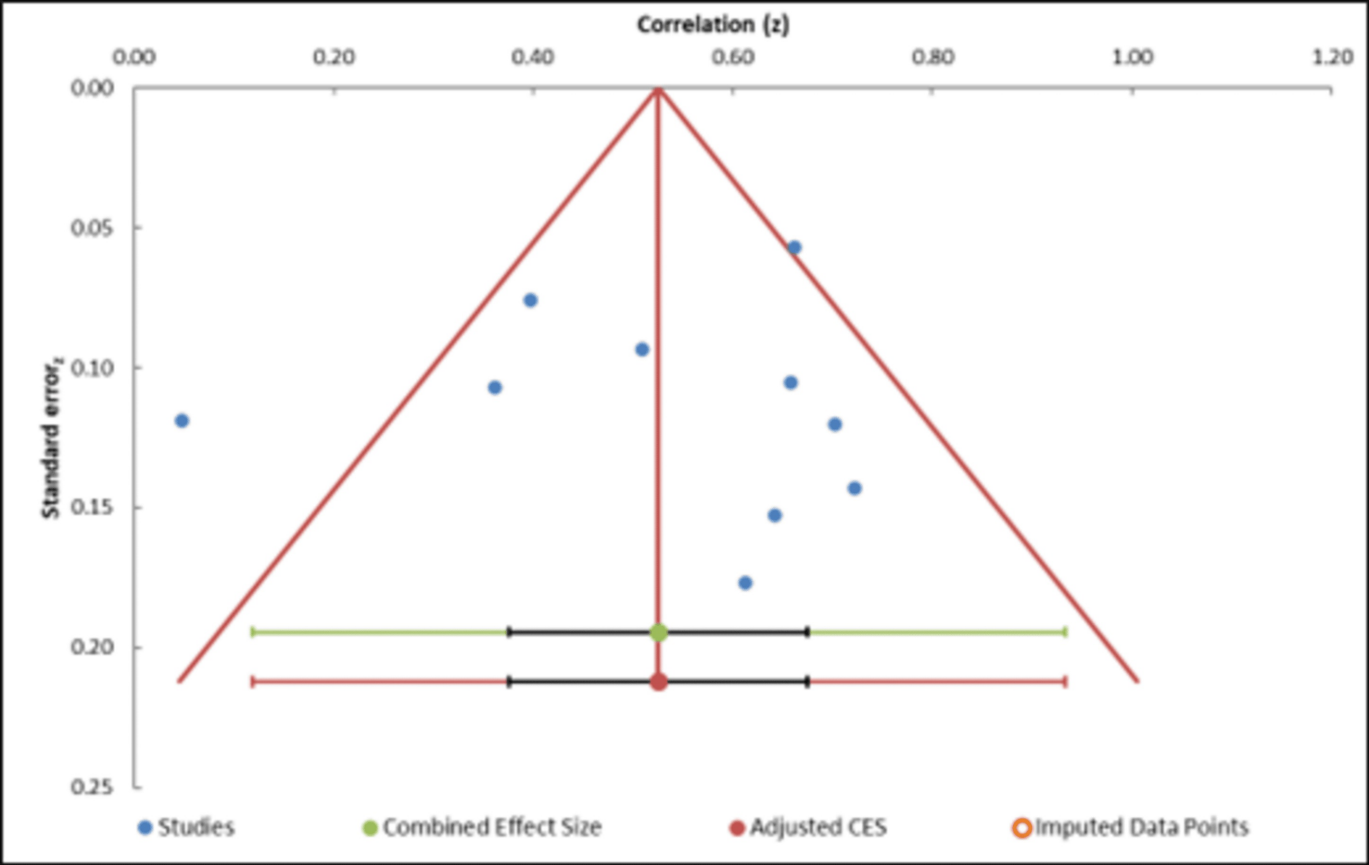
Funnel plot. CES: combined effect size.

The funnel plot analysis assesses the presence of publication bias (Figure 4). The plot shows a relatively symmetric distribution of studies, suggesting minimal publication bias [28]. Egger’s regression test (Table 5) yielded an intercept of 1.62 (p = 0.68), indicating no statistically significant small-study effects. Additionally, the trim-and-fill method did not identify any missing studies, further supporting the absence of substantial publication bias. Despite some dispersion in effect sizes, the results suggest that publication bias is unlikely to influence the meta-analysis findings significantly [29].

**Table 5:** Egger regression.

		Egger regression		
	Estimate	Standard error	Confidence interval, lower limit	Confidence interval, upper limit
Intercept	1.62	3.75	-6.87	10.11
Slope	0.20	0.76	-1.51	1.91
t test	0.43			
p-value	0.68			

Meta-analysis findings

Forest Plot

**Figure 5:**
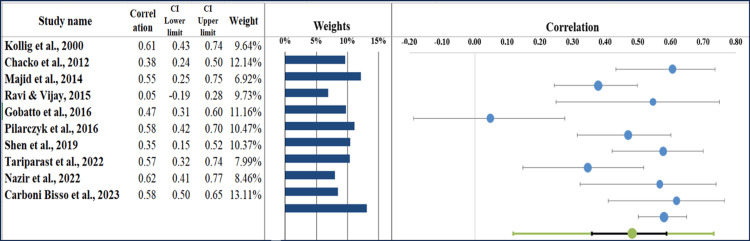
Forest plot showing individual study correlation estimates and the pooled correlation under a random-effects model. Kollig et al. (2000) [16], Chacko et al. (2012) [17], Majid et al. (2014) [18], Ravi & Vijay (2015) [19], Gobatto et al. (2016) [20], Pilarczyk et al. (2016) [21], Shen et al. (2019) [22], Tariparast et al. (2022) [23], Nazir et al. (2022) [24], Carboni Bisso et al. (2023) [25].

The meta-analysis found a pooled correlation of r = 0.48 (95% CI: 0.41 to 0.55), indicating a moderate positive relationship between the studied variables. The forest plot (Figure 5) displays individual study estimates, demonstrating variability in correlation strengths across included studies. Among the included studies, Carbon Bisso et al. [25] reported one of the strongest correlations (r = 0.58, 95% CI: 0.50-0.65), highlighting a significant positive association. Similarly, Nazir et al. [24] and Pizarczyk et al. [21] showed correlations of r = 0.62 (95% CI: 0.41-0.76) and r = 0.58 (95% CI: 0.43-0.74), respectively, further supporting the relationship. In contrast, Ravi & Vijay [19] reported a weaker correlation (r = 0.05, 95% CI: 0.18-0.28), suggesting potential methodological differences or population variability. Additionally, Chacko et al. [17] and Tariparast et al. [23] found correlations of r = 0.38 (95% CI: 0.25-0.50) and r = 0.57 (95% CI: 0.32-0.74), showing moderate but significant associations [30].

Heterogeneity Assessment

The heterogeneity assessment of the meta-analysis revealed substantial variability among the included studies (Table 6). High Q statistic (Q, 33.27; p < 0.001) showed that the observed variance of correlations is much higher than what chance would predict. Heterogeneity was high, with a considerable amount of I² value (72.55%). Thus, a substantial amount of variability in effect sizes was found to be due to differences in studies rather than solely random error. Also, the T² value (0.03) indicated a significant between-study variance, and the T value (0.18) amounted to a moderate range of effect sizes. These findings support the idea that the heterogeneity recorded in these studies may be due to methodological dissimilarities, such as differences in participant base, diagnostic standards, and assessment methods [31].

**Table 6:** Information related to the forest plot.

Meta-analysis model	
Model	Random effects model
Confidence level	95%
Combined effect size	
Correlation	0.48
Confidence interval, lower limit	0.36
Confidence interval, upper limit	0.59
Prediction interval, lower limit	0.12
Prediction interval, upper limit	0.73
Z-value	8.03
One-tailed p-value	0.000
Two-tailed p-value	0.000
Number of incl. subjects	1069
Number of incl. studies	10
Heterogeneity	
Q	33.27
p_Q_	0.000
I^2^	72.95%
T^2 ^(z)	0.03
T (z)	0.17

Subgroup Analysis

**Figure 6:**
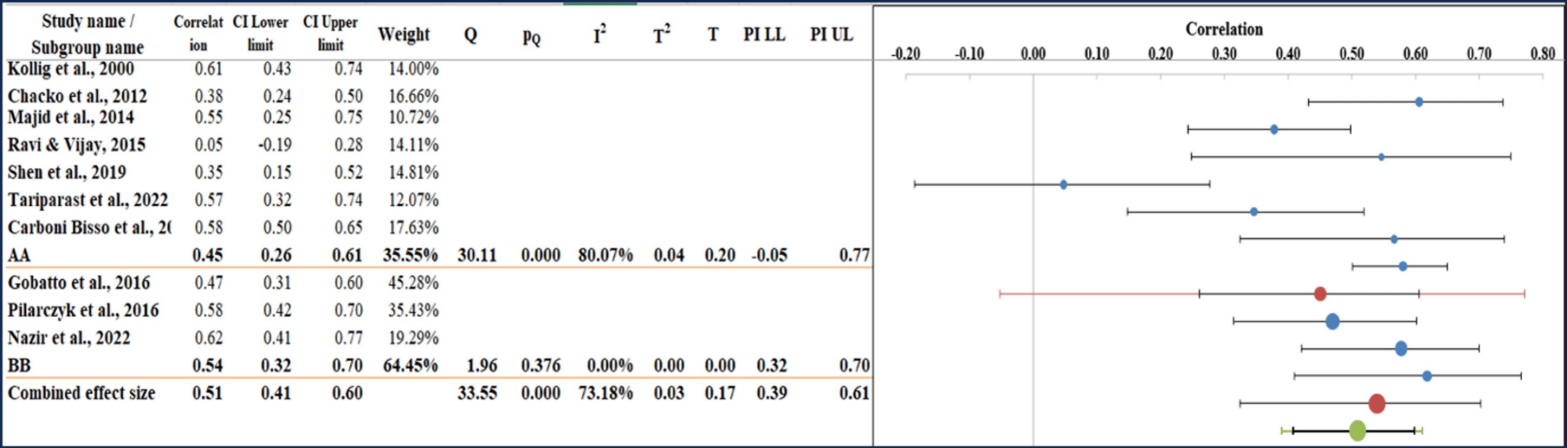
Subgroup analysis of the included studies showing pooled correlation estimates for procedural success and complications in USPDT and BPDT, stratified by patient characteristics, study design, and operator expertise. Kollig et al. (2000) [16], Chacko et al. (2012) [17], Majid et al. (2014) [18], Ravi & Vijay (2015) [19], Gobatto et al. (2016) [20], Pilarczyk et al. (2016) [21], Shen et al. (2019) [22], Tariparast et al. (2022) [23], Nazir et al. (2022) [24], Carboni Bisso et al. (2023) [25]. USPDT: ultrasound-guided percutaneous dilatational tracheostomy; BPDT: bronchoscopy-guided percutaneous dilatational tracheostomy; CI: confidence interval; PI: prediction interval; LL: lower limit; UL: upper limit.

The overall combined effect size was 0.53 (95% CI: 0.35-0.67), which indicates a moderate positive correlation. The analysis was split into two main subgroups. Subgroup A showed correlation values ranging from 0.05 to 0.61, while subgroup B exhibited a slightly higher range from 0.55 to 0.61, suggesting some variability between the groups. Despite this categorization, heterogeneity remained substantial (I² = 72.95%), indicating that subgrouping alone did not fully explain the variation in study results. The Q statistic (4.41, p = 0.056) suggests that additional factors, such as differences in gastroesophageal reflux disease (GERD) diagnostic criteria, population characteristics, and assessment methods, may contribute to the observed heterogeneity (Figure 6 and Table 7) [32].

**Table 7:** Subgroup analysis of the pooled effect sizes for procedural success and complications in USPDT and BPDT, categorized by patient characteristics and study design. USPDT: ultrasound-guided percutaneous dilatational tracheostomy; BPDT: bronchoscopy-guided percutaneous dilatational tracheostomy.

Meta-analysis model			
Combined effect size			
Correlation	0.53		
Confidence interval, lower limit	0.35		
Confidence interval, upper limit	0.67		
Prediction interval, lower limit	0.23		
Prediction interval, upper limit	0.74		
Number of incl. subjects	1069		
Number of incl. studies	10		
Number of subgroups	2		
Analysis of variance	Sum of squares (Q*)	Df	P
Between/Model	4.41	1	0.036
Within/Residual	7.20	8	0.515
Total	11.61	9	0.236
Pseudo R^2^	37.98%	-	-

Discussion

This systematic review and meta-analysis provide a comprehensive evaluation of the integration of USPDT and BPDT, emphasizing their synergistic potential in optimizing procedural safety, precision, and clinical outcomes. While both modalities have individually demonstrated substantial efficacy - ultrasound offering superior anatomical visualization of vascular and soft tissue structures, and bronchoscopy ensuring precise intraluminal guidance and real-time confirmation of tracheal entry - their combined application remains an underutilized yet highly transformative approach [33]. Together, meta-analysis findings confirm that USPDT and BPDT contribute significantly to improved procedural success with different complication rates and clinical applicability between patients in other populations.

Both techniques have moderate positive correlation values in the pooled effect size of the meta-analysis and favor the technique's effectiveness in PT. Specifically, the findings show that USPDT has a significant impact on lowering bleeding risks and vascular complications, particularly in high-risk patients such as obese, coagulopathic patients. In contrast, BPDT offers better real-time airway visualization with accurate tracheal entry and fewer than 1% of posterior tracheal wall injuries. Evidence from individual studies, such as Ravi & Vijay [19] and Nazir et al. [24], confirms that USPDT results in lower hemorrhage rates and shorter procedural durations, Gobatto et al. [20] and Shen et al. [22] support BPDT in lowering complications associated with airways and improving the first pass success.

Although both techniques have had high success rates, there was heterogeneity in outcomes. Variation in complication rate and procedural duration was likely a function of patient selection, operator experience, and institutional protocols. Carboni Bisso et al. [25] also reported that the risk of oxygen desaturation is greater in COVID-19 patients than in healthy individuals when receiving BPDT, which is associated with patient physiology. Tariparast et al. [23] also reported the impact of single-use disposable bronchoscopes, which are non-inferior to reusables, and cost-related challenges.

The results drawn from this review agree with previous studies that investigated whether ultrasound and bronchoscopy played a part in PT [33-35]. Existing literature supports that USPDT reduces procedural complications by enhancing pre-procedural anatomical assessment, minimizing vascular injuries, and decreasing overall bleeding risks [36]. These results are consistent with meta-analyses that have previously suggested that USPDT is particularly beneficial in patients with complex airway anatomy, where blind puncture techniques pose a higher risk [36-38].

On the other hand, BPDT has been widely endorsed in the literature for its superior visualization capabilities, with studies emphasizing its role in preventing tracheal misplacement and reducing the risk of subglottic stenosis [34]. However, concerns about prolonged procedural durations and the requirement for bronchoscopy-trained personnel remain standard limitations cited in previous reviews. This systematic review and meta-analysis further corroborate these findings, demonstrating that while BPDT provides essential airway guidance, it may not be as time-efficient as USPDT in routine clinical practice [35].

Limitations of the Study

One limitation of this systematic review and meta-analysis is that the protocol was not registered with PROSPERO (International Prospective Register of Systematic Reviews), a standard practice to enhance transparency and reduce the risk of bias. While efforts were made to adhere to the PRISMA guidelines, the lack of protocol registration may impact reproducibility and methodological rigor. The dual-modality approach, combining ultrasound and bronchoscopy for PT, has yet to achieve widespread implementation, as most clinical practices and studies have focused on using either method in isolation. Bronchoscopy has been predominantly favored for its ability to provide direct visualization of the tracheal lumen, enabling precise tube placement. At the same time, ultrasound is highly regarded for its capacity to visualize superficial anatomical structures and vascular landmarks. It is a real-time visualization of the needle from the skin to the anterior wall, thereby minimizing the risk of vascular injury. However, the limitations of these standalone techniques are well-documented. Bronchoscopy alone cannot identify vascular structures external to the trachea, which can increase the risk of bleeding, while ultrasound, although effective in mapping external anatomy, may fail to reliably confirm tracheal entry or ensure accurate tube positioning, particularly in patients with distorted anatomy or in emergent situations. The limited adoption of the dual-modality approach is likely due to practical and logistical challenges, including the need for specialized equipment, the coordination of two imaging modalities during the procedure, and the requirement for trained personnel proficient in their simultaneous application. However, as all studies were highly heterogeneous due to various designs, patient selection, and procedural protocols, direct comparisons could not be made. Variability of the operator's expertise, institutional preferences, and equipment availability also influence outcomes. Standardization is limited, and confusion about what is reported compounds the generalizability of findings and emphasizes the importance of multicenter trials to define uniform best practices. However, the approach has an apparent advantage through these barriers since it is synergistic in relieving the individual weaknesses of each technique to enhance procedural safety, precision, and patient outcomes. This emphasizes the need for more attention to be paid to the implementation of surgical tracheostomy in clinical practice, as it has the potential to significantly improve the standard of care in complex and high-risk tracheostomy cases.

Future Directions

Current literature does not explore the dual modality approach combining ultrasound and bronchoscopy during PT. Although each modality is documented to provide benefits when practiced individually, the synergistic integration of the modalities addresses critical deficiencies. It is an essential device to obtain the best result, especially in obese patients, patients with distorted neck anatomy, and critically ill patients who require close attention. In the future, large-scale multicenter randomized controlled trials should protect the use of this combined approach safely, effectively, and cost-effectively. On the same token, it is time to establish standardized procedural protocols and standard clinical training to implement universally in diverse clinical settings.

Furthermore, AI-assisted imaging and combined ultrasound-bronchoscopy units could be used to make the procedure possible in resource-constrained settings. It is impossible to estimate how quickly this technique must be used in routine care to establish a new bar of care and improve results in PT procedures. Only a few studies have been performed on this dual-modality technique and its apparent merits. To control for heterogeneity in the meta-analysis, standardization of study design, inclusion criteria, and procedural technique is required in future research. In addition, clinical implementation requires studies of cost-effectiveness and structured training indices. Investigating AI-assisted imaging and hybrid integration strategies could further optimize safety, efficiency, and accessibility across diverse healthcare settings.

## Conclusions

This systematic review and meta-analysis confirm that ultrasound-guided and bronchoscopy-guided percutaneous tracheostomy techniques are highly effective, offering unique advantages. USPDT enhances vascular mapping and minimizes bleeding risks, while BPDT provides superior airway visualization and ensures accurate tracheal entry. Integrating both techniques may represent the optimal approach for maximizing procedural safety and efficiency, particularly in high-risk populations. Future research should focus on large-scale trials to further define best-practice guidelines and improve the standardization of procedural protocols across diverse clinical settings.
